# Multidimensional Internet Use, Social Participation, and Depression Among Middle-Aged and Elderly Chinese Individuals: Nationwide Cross-Sectional Study

**DOI:** 10.2196/44514

**Published:** 2023-08-30

**Authors:** Xiwang Du, Jiazhi Liao, Qing Ye, Hong Wu

**Affiliations:** 1 Taikang Tongji (Wuhan) Hospital Wuhan China; 2 Tongji Hospital Tongji Medical College Huazhong University of Science and Technology Wuhan China; 3 School of Medicine and Health Management Tongji Medical College Huazhong University of Science and Technology Wuhan China

**Keywords:** internet use, depression, social participation, middle-aged and elderly Chinese, RIDL

## Abstract

**Background:**

There is growing evidence that the internet has beneficial effects on the mental health of middle-aged and older people (≥45 years), but the evidence is inconclusive, and the underlying mechanisms are less known.

**Objective:**

This study aims to explore the relationship between multidimensional (devices, frequency, and purpose) internet use and depression in middle-aged and elderly Chinese, as well as the mediating effect of social participation. Moreover, this study will explore the moderating effect of the regional informatization development level (RIDL) on the relationships between individual internet use, social participation, and depression.

**Methods:**

Data on 17,676 participants aged 45 years or older were obtained from the China Health and Retirement Longitudinal Study (CHARLS) 2018 data set. The 10-item Center for Epidemiologic Studies Depression Scale (CES-D-10) was used to identify the presence of depression. Logistic regression was used to explore the relationship between each dimension of internet use and depression. Multiple linear regression was used to explore the mediating effect of social participation and the moderating effect of the RIDL.

**Results:**

The results showed that 28.33% (5008/17,676) of the total population had depression. In terms of regional subgroups, respondents living in the western region exhibited the highest proportion of depression (2041/5884, 34.69%). Internet use was negatively associated with depression (odds ratio 0.613, 95% CI 0.542-0.692; *P*<.001). Various dimensions of internet use positively contributed to individual social participation and reduced individual depression (devices: β=–.170, 95% CI –0.209 to –0.127; frequency: β=–.065, 95% CI –0.081 to –0.047; and purpose: β=–.043, 95% CI –0.053 to –0.031). In addition, the RIDL weakened the relationship between individual-level internet use and social participation (internet use: *F*_74.12,9.82_=7.55, *P*<.001; devices: *F*_51.65/9.88_=5.23, *P*=.005; frequency: *F*_66.74/10.08_=6.62, *P*=.001; and purpose: *F*_66.52/9.78_=6.80, *P*=.001), and negatively moderated the relationship between the frequency of internet use and depression (frequency: *F*_662.67/188.79_=3.51, *P*=.03).

**Conclusions:**

This study found that different dimensions of internet use are associated with lower levels of depression. Social participation partially mediates the association between multidimensional internet use and depression in the eastern, central, and western regions, respectively. Additionally, the RIDL helps individuals further their internet use and social participation, reducing the impact of depression. However, this effect weakens sequentially from the western region to the central region and then to the eastern region.

## Introduction

### Background

Depression, a prevalent chronic mental disorder associated with a high incidence, disability, suicide risk, and disease burden, has progressively emerged as a significant public health challenge, posing threats to both the physical and mental well-being of individuals [1]. Depression impairs human capital and is associated with premature mortality from suicide and other illnesses [2]. A range of psychological therapies, including antidepressant medications, transcranial magnetic stimulation, modified electroconvulsive therapy, and deep brain stimulation, may be effective to reduce depression [3-5]. However, about one-third of people with depression have failed to respond to traditional antidepressant treatment [6]. Moreover, these interventions have not reached many of those in need in low-, middle-, or high-income countries [7], further contributing to the global burden of the disease.

China is the most populous country globally and one of the countries with the fastest aging population. The aging of the population is accompanied by a rapidly increasing prevalence of depression. According to a report by the World Health Organization, there are more than 54 million patients with depressive disorder in China [8], accounting for approximately 17% of the global burden of mental disorders [9], and the lifetime prevalence of depressive disorder in Chinese adults is 6.8% [10]. In addition, in China, the proportion of middle-aged and elderly people aged 45 years and older suffering from depression is quite high [11], and the overall prevalence of depression in the elderly population (age >60 years) ranges from 11% to 57% [12]. Predictions indicate that the economic burden and stigma caused by depression will continue to increase in China [13]. Therefore, addressing China’s problems may go a long way toward improving global mental health.

Despite China’s transition into the ranks of upper-middle-income countries, it continues to grapple with the significant issue of an inadequate supply of professional resources, even as the demand for mental health services is on the rise. Currently, there are fewer than 8.75 mental health workers per 100,000 inhabitants in China’s mental health system [14], which is slightly higher than the average in low- and middle-income countries but much lower than that of high-income countries [15]. The profound gap between need and supply in mental health services indicates that novel auxiliary solutions with greater accessibility are urgently needed [16].

As a tool across time and space, the internet can be used in many ways, such as searching for health information, following online fitness, entertainment, and remote contact with family and friends. These functions could help older adults overcome some physical and functional limitations and provide benefits for their health and well-being. Therefore, it has the potential to reduce loneliness as well as reduce depressive symptoms, thereby promoting mental health. Several empirical studies have explored the relationship between internet use and depressive symptoms in middle-aged and older adults [17-20]; however, based on a review of the existing literature, we found that there exist the following research gaps. First, only a few studies have further explored the mechanisms and pathways between internet use and depressive symptoms, especially the role of social participation. Second, existing research mainly focuses on analyzing internet usage at the individual level among middle-aged and elderly populations, and it lacks an exploration of the influence mechanisms of the regional informatization development level (RIDL) at this individual level. Third, research findings are inconsistent. Some studies have shown that the use of the internet in older groups can enhance their contact with the outside world, expand the scope of communication, increase social support, and avoid social isolation, which can reduce depression levels and thus have a positive impact on mental health [21-23]. Some studies have expressed a diametrically opposite view that internet use will occupy the social time of the user group and reduce the opportunities to conduct offline face-to-face communication, which is not conducive to the maintenance of emotional expression and social connection and may promote the emergence of depressive symptoms [24-26]. Therefore, existing studies do not agree on the relationship between dimensions of internet use, such as terminal device, frequency, and purpose and depressive symptoms. Fourth, previous studies have paid more attention to the urban-rural digital divide [27,28]. However, the regional development of information technology in China is extremely uneven. The Information Technology Development Index in Eastern China is notably higher, showing a decreasing trend from the eastern to central and western regions. The largest differences in development index values, including the highest and lowest indexes, are observed between the east and the west, resulting in a significant regional disparity in internet usage levels.

### Study Objective

**Figure 1:**
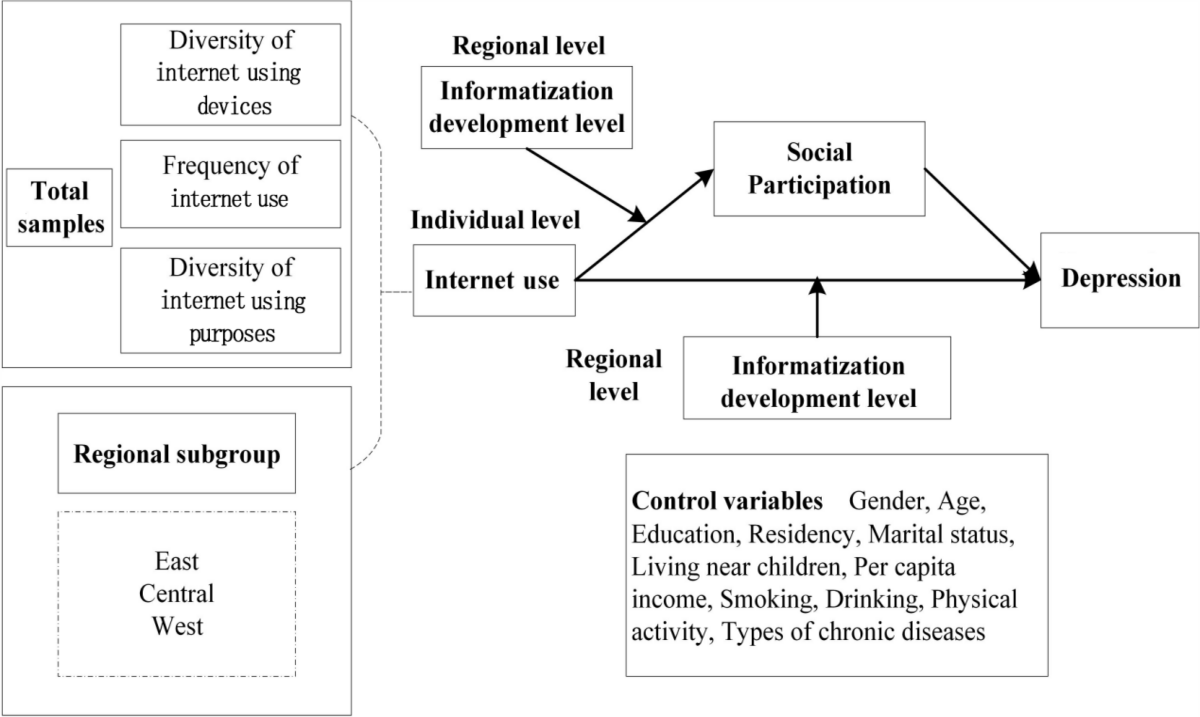
The conceptual framework.

Based on the above, our study first analyzes the relationship between multidimensional internet use and depressive symptoms in Chinese middle-aged and older people using the latest publicly available data from the only large database representative of middle-aged and older people in China. This study also measures the association between individual-level multidimensional internet use, social participation, and its effects on depressive symptoms. On this basis, this study will investigate whether the RIDL moderates the relationship between individual multidimensional internet use and social participation and depressive symptoms. In addition, this study will reveal potential heterogeneous influences for different regional subgroups in the eastern, central, and western regions by combining them with China’s national background. The conceptual framework of this study is shown in Figure 1.

### Literature Review

#### Internet Use and Depression

Researchers are increasingly concerned about the impact of internet use on mental health, such as depressive symptoms. In this regard, extensive research and exploration of the relationship between internet use and mental health have been conducted.

However, previous studies that attempted to analyze the relationship between internet use and depression yielded different results. Some studies have indicated that internet use can easily lead to internet addiction, resulting in a decline in social participation, interpersonal relationships, and overall mental health. This effect is particularly pronounced among teenagers [29]. One study showed that both the time people spent browsing the web at home and the time they spent on other internet interactions (eg, instant messaging exchanges, chat rooms, and news) were both negatively related to life satisfaction and positively related to loneliness [30]. Excessive use of the internet can also lead to addiction and feelings of loneliness [31]. Furthermore, excessive interaction on social networks, constant social comparisons, and privacy invasions can trigger negative emotions [32], potentially leading to depression and anxiety [33].

In studies conducted among middle-aged and elderly individuals, some scholars believe that older adults use the internet to seek technological support, improve their mood through engaging in distracting activities, normalize their experiences, and express their mental states [34]. The frequency of using the internet to contact family members can mediate the relationship between internet use and self-rated subjective well-being [35]. Internet use had a protective effect on relieving depression, and it can increase the positive effect of social relationship satisfaction in the elderly, thereby alleviating depression [36]. In assisted and independent living communities, internet use may also help reduce loneliness and increase social participation in older adults [37]. In addition, one study [38] showed that internet terminal device use, mobile phone use, and occasional internet use in middle-aged and older adults were associated with a lower risk of depression. Other studies have found that internet use has a positive contribution to mental health and reduces depression classifications by 20%-28% [39]. However, there are also studies that reveal the opposite result. They argue that increased time spent online erodes older people’s social interactions, leading to reduced social engagement, diminished social networks, and even negative internet interactions and social comparisons, which in turn worsen older people’s mental health. The negative effects are particularly pronounced among specific groups of people [28,40-42].

Although some studies have further investigated the effects of specific devices (eg, mobile phones, computers, or tablets) on mental health [41,42], they have failed to agree on the optimal device for internet use. Regarding the frequency of internet use, some studies have shown that a higher frequency of use is associated with a more positive effect on mental health [43], while other studies suggest that occasional use may alleviate depression more effectively [38]. For the purpose of internet use, the internet as a tool can be used in many ways, such as searching for health information, following online fitness, entertainment, and connecting with family and friends remotely. However, the existing studies mostly focus on a single type of internet activity [44-46].

#### The Mediating Role of Social Participation: Structural Social Capital Perspective

Social capital mainly refers to the social network and resources within the group or local community, including trust, social contact, and social participation, among others [47]. From the attribute level, social capital is usually composed of cognitive social capital (eg, trust) and structural social capital (eg, social network and social participation) [48]. Middle-aged and elderly individuals often encounter numerous stressful events, which can stem from physical ailments or social disparities. Those who face these challenges may receive additional support through social capital to mitigate these negative effects, ultimately enhancing their mental health.

Internet use can impact the structural aspects of social capital. On the one hand, this decline can be attributed to various factors, such as the diminishing demographic dividend, widespread labor migration, and the integration of urban and rural areas. Consequently, coresidence between Chinese parents and adult children is becoming less common. This trend has led to an increasing number of middle-aged and elderly individuals living alone, potentially resulting in a sense of detachment from their adult children. Such circumstances could contribute to a reduction in structural social capital, which may affect their physical and mental health. Compared with traditional communication channels, the internet has the advantages of convenience, connectivity, and widespread accessibility. Prior studies have demonstrated that the internet serves as a valuable tool for seniors, enabling them to maintain connections with distant family and friends. This increased connectivity via the internet has been associated with higher levels of interaction with adult children, leading to enhanced well-being among older individuals in China, ultimately contributing to improved mental health [46,49].

Drawing from the framework of structural social capital, internet use has the potential to increase the social participation of middle-aged and elderly people. As individuals age and encounter shifts in social roles, middle-aged and elderly individuals often encounter challenges such as the gradual reduction of their social circles and difficulties in sustaining interpersonal relationships [50]. These shifts frequently contribute to increased feelings of loneliness and social isolation, rendering them more susceptible to experiencing depressive symptoms. The internet offers a valuable opportunity for them to transition from social avoidance to active social participation [51], which enables them to access many social opportunities, both online and offline, through tools (eg, smartphones). They can effortlessly exchange information, share life events, or photos through online activities and various platforms, such as social networking sites or portals (eg, Weibo). They can also discover clubs, organizational activities, and attend local religious events (eg, gatherings and volunteering) [52]. This expansion and strengthening of social relationships [53] can enhance self-identity and foster a sense of belonging, ultimately slowing down the pace of social disengagement [54]. These positive effects are beneficial for the improvement of depressive symptoms [55]. Additionally, the convenience of internet technology facilitates advanced communication through instant messaging, enabling people to coordinate their participation in activities, decide on timing, and choose meeting places, among other things. This convenience also extends to traditional Chinese social activities, such as playing mahjong/chess and engaging in public square dancing, all of which help increase social interactions, ultimately leading to a reduction in depressive symptoms [56]. Diverse and beneficial internet usage can enhance social participation among middle-aged and elderly individuals. However, online social engagement does not substitute for offline participation; rather, it only broadens the spectrum of activities, encompassing both virtual and physical realms [57].

#### External Environment: Regional Information Development Level

China is a typical country with uneven geographical development, and the RIDL is extremely unbalanced between regions. The eastern region, primarily coastal and relatively flat, has experienced an early onset of economic development, reaching a high level of prosperity. These areas also display greater openness to the global community and prioritize the advancement of information technology. By contrast, the central and western regions, especially the latter, are often sparsely populated with relatively poor economic development. Consequently, these areas have a low level of investment in the development of the information industry and the construction of information technology infrastructure. For example, there is a significant difference between the Yangtze River Delta, representative of the eastern region, and the lagging regions in the central and western parts of the country. These regional imbalances in information technology development have resulted in the following effects.

Although information and communications technology has rapidly developed, middle-aged and elderly people face various factors such as personal attributes (eg, lack of interest in learning and self-efficacy), functional limitations (eg, decreased memory or logical analysis), structural constraints (eg, high cost and poor design), and interpersonal constraints (eg, a lack of initiation and use support). These inequalities in access and use prevent them from fully exploring opportunities and participating in society, making them vulnerable [58-60]. These restrictions also apply to middle-aged and elderly groups. Regions with higher levels of informatization development generally have better internet penetration, broadband service quality, high-speed network infrastructure, development environment, industrial digitization, and information technology industries. Additionally, residents in this area typically exhibit higher cognitive abilities, education levels, and socioeconomic statuses, leading to relatively good information technology literacy and proficiency. Even with the same frequency of internet use, residents in high-informatization areas tend to achieve higher utilization efficiency, resulting in a more effective transformation of social participation. Therefore, the impact of internet use on social participation is greater for residents in high-informatization areas.

Digital health literacy and internet connectivity have recently been recognized as “super social determinants of health” [61] due to their significant impact on broader determinants of health. In the context of the COVID-19 pandemic, they have also been referred to as “social vaccines,” empowering individuals and communities to combat the spread of misleading disease information by accessing and utilizing virus-related information [62]. However, several factors, such as age, gender, socioeconomic status, health status, and urban or rural living environments, can influence the development of health and digital literacy. Residents in areas with advanced information development may have higher health information literacy, leading to broader exposure and awareness of depressive symptoms. Web-based resources can support them in enhancing their knowledge and self-management. Therefore, the impact of internet use on depressive symptoms may be more significant for residents in high-informatization areas.

## Methods

### Sample and Data Collection

The China Health and Retirement Longitudinal Study (CHARLS) is a large-scale, nationally representative population tracking survey conducted in 28 provinces across China. This survey employs a multistage probability sampling method to gather comprehensive data on various aspects of middle-aged and elderly individuals’ lives. To ensure the best practices and international comparability of results, the CHARLS team collaborates with leading international studies in the field of health and retirement research to create a high-quality public database. The survey captures extensive information, including household demographics, health status, health care utilization, insurance coverage, and other variables essential for scientific research aimed at serving the needs of the middle-aged and elderly population [63]. Given the rapid evolution of internet usage, this study utilized data from the latest wave 4 survey conducted in 2018, which encompassed 19,816 respondents. For the investigation of the association between internet use and depression, we selected 17,676 respondents based on the following criteria: (1) providing information about both internet use and the 10-item Center for Epidemiologic Studies Depression Scale (CES-D-10) score, and (2) being aged 45 and above. The age of 45 years is typically considered the cut-off point for middle-aged individuals in China. At this age, people often begin a transition toward semiretirement, which marks a significant life phase change. Additionally, according to the life-cycle theory, physical function tends to decline after the age of 45 years, signifying the gradual entry into the declining phase of the life cycle. Furthermore, the trajectory of depression is influenced by age, and the prevalence of depression exhibits an inverted U-shaped distribution. Individuals in the middle age (aged ≥45 years) and older age groups are more susceptible to depression due to specific physiological and social factors, with the peak prevalence occurring between the ages of 55 and 74 years.

### Ethical Considerations

Before conducting the CHARLS survey, well-trained interviewers informed each participant about the survey’s content, and each interviewee signed an informed consent form. The survey content is strictly confidential and all data about respondents are protected by data security and privacy laws. The Ethics Review Committee of Peking University has provided ethical approval for all waves of the CHARLS survey (ethical approval number IRB 00001052-11015).

### Variables

#### Depression

In the CHARLS 2018 questionnaire, researchers used the CES-D-10 to assess depression among respondents. The 10 items primarily pertain to the feelings and behaviors of the interviewees in the previous week. The answers on the CES-D-10 were scored on a 4-level scale: 0=little or no (<1 day), 1=not much (1-2 days), 2=sometimes or half of the time (3-4 days), and 3=most of the time (5-7 days). The respondents’ depressive scores range between 0 and 30. A lower score indicates a lower level of depression, and vice versa. Studies have shown that the CES-D-10 has good validity and reliability [64] among the middle-aged and elderly Chinese population. Additionally, in previous studies, a threshold of 12 was typically used to identify depression, and it has been demonstrated that this threshold effectively identifies clinically significant depression [56,65,66]. Therefore, the study established a dichotomous variable at the 12-point cut-off to determine whether respondents had depression (0=no, 1=yes), considering those with a score greater than or equal to 12 as having depression. The CES-D-10 scores were used in mediating and moderating analyses to examine changes in depression under different mechanisms of action.

#### Individual-Level Internet Use

In the CHARLS 2018 questionnaire, the following questions were used to measure internet use: (1) Have you used the internet in the last month? (2) If yes, which types of devices do you use to access the internet? (3) How often in the last month? (4) What do you usually do on the internet? (5) Do you use mobile payments, such as Alipay or WeChat Pay? (6) Do you use WeChat? (7) Do you post WeChat moments?

This study intends to measure the internet use of middle-aged and elderly people in China from the following dimensions: diversity of internet use devices, frequency of internet use, and diversity of internet use purposes (Textbox 1).

**Dimensions used to measure internet use of middle-aged and elderly people in China.**

**1. Diversity of internet use devices**

This study defines this variable based on 4 groups: desktop computers, laptop computers, tablets, and mobile phones. Each item used in the study is counted as 1 point, and the total score ranges between 0 and 4 points. A higher score indicates greater diversity in internet use devices.

**2. Frequency of internet use**

In the CHARLS 2018 survey, respondents were asked about the frequency of their internet use, with options ranging from “almost every day” to “almost every week” to “not often.” For this variable, the study used reverse encoding, with the following coding: 1=infrequently, 2=almost every week, and 3=almost every day.

**3. Diversity of internet use purposes**

In this study, the variable is defined across 8 categories: chatting, watching news, watching videos, playing games, financial management, mobile payment, WeChat, and moments. Each item in the study is assigned 1 point, resulting in a total score between 0 and 8 points. A higher score indicates a broader range of purposes for internet use.

#### Regional Information Development Level

This study obtained relevant information on the development level of informatization from the “Digital China Development Report” [67] issued by the Cyberspace Administration of China and utilized it as a variable for the RIDL. Reverse coding was applied to this variable, with the first, second, and third echelons coded as 3, 2, and 1, respectively. Detailed information is provided in Table 1.

**Table 1:** The rank of the regional informatization development level.

Ranking	Province (autonomous region/municipality)
The first echelon	Beijing, Zhejiang, Shanghai, Guangdong, Jiangsu, Tianjin, Shandong, Hubei, Sichuan, Fujian
The second echelon	Chongqing, Anhui, Henan, Jiangxi, Hunan, Shaanxi, Hebei, Liaoning, Guizhou
The third echelon	Guangxi, Shanxi, Jilin, Yunnan, Inner Mongolia, Heilongjiang, Gansu, Xinjiang, Qinghai

#### Social Participation

For the measurement of social participation, we selected 7 types of social participation activities from the CHARLS 2018 data set. We excluded types of social participation that constituted less than 0.5% of the participating samples. The selected activities were as follows: (1) visiting or socializing with friends; (2) playing mahjong, chess, cards, or participating in community room activities; (3) offering help to noncohabiting relatives, friends, or neighbors; (4) engaging in activities such as dancing, fitness, or practicing qigong; (5) participating in community events; (6) involvement in volunteer or charity activities; (7) providing care for sick or disabled individuals not living with the respondent. Each chosen type of social participation is assigned a point on a scale from 0 to 7, with a higher score indicating a greater level of social participation among the respondents.

#### Control Variables

Based on previous studies, this study considered potential confounding variables associated with internet use and depressive symptoms, categorizing these control variables into 3 groups. Physical activity was primarily measured based on whether respondents engaged in at least 10 minutes of high-, moderate-, or low-intensity physical activity per week. The count of chronic diseases served as the primary measure of respondents’ chronic health conditions. The specific assignment instructions for each covariate are detailed in Table 2.

**Table 2:** Control variable assignment description.

Variable		Definition/codes
**Sociodemographic variables**		
	Gender	1=male; 2=female
	Age	Continuous variable
	Residency	1=urban; 2=rural
	Education	1=illiterate; 2=primary school or below; 3=middle school; 4=high school or above
	Marital status	1=single (single/divorced/widowed); 2=partnered (married/partnered)
	Retirements	0=no; 1=yes
	Live near the children	0=no; 1=yes
	Per capita household income	Total household income/square root (number of family members)
**Health behavior variables**		
	Smoking	0=no; 1=yes
	Drinking	0=no; 1=yes
	Physical activity	0=no; 1=yes
**Health outcome variable**		
	Chronic diseases^a^	0=none; 1=1 type; 2=2 types; 3=3 or more types

^a^Chronic diseases are hypertension, hyperlipidemia, hyperglycemia, malignant tumors, chronic lung disease, liver disease, heart disease, stroke, kidney disease, stomach disease, arthritis, asthma, mental problems, and memory-related diseases.

### Data Analysis

Statistical analysis was conducted using R Studio (The R Foundation for Statistical Computing). First, we performed descriptive statistics on the basic characteristics of the variables. The categorical variables were represented by frequency (n) and percentage (%). We checked the distribution of continuous variables and found that the kurtosis and skewness of age were both less than 1, indicating a normal distribution. However, per capita household income did not conform to the normal distribution, so we applied a logarithmic transformation. Continuous variables following a normal distribution were represented by mean and SD, while nonnormally distributed continuous variables were represented by the median. The Kruskal-Wallis test was employed to analyze the basic characteristics of subgroups in the eastern, central, and western regions. Subsequently, logistic regression was used to explore the relationship between each dimension of internet use and depression. Based on this, multiple linear regression was applied to investigate the mediating effect of social participation on the relationship between individual-level internet use and depressive scores. Additionally, we examined the moderating effects of the RIDL on individual-level internet use, social participation, and depressive scores. We utilized the “Bruce” package in R for conducting the mediating and moderating effect analyses. The bootstrap method was adopted with 1000 random sampling iterations for the mediating effect test. Variables were standardized before data analysis.

## Results

### Descriptive Statistics

Table 3 presents the sample characteristics. Of the 17,676 respondents, the majority lived in rural areas (n=10,658, 60.30%), and most of them were female (n=9243, 52.29%). A significant proportion had a primary school education or below (n=12,187, 68.95%; *P*<.001). The majority of respondents were partnered (n=15,237, 86.20%). Additionally, a substantial number of respondents did not consume alcohol (n=11,622, 65.75%) or smoke (n=12,919, 73.09%). Most engaged in physical activities (n=16,126, 91.23%) and had been diagnosed with 1 or more chronic diseases by doctors (n=14,099, 79.76%).

**Table 3:** Sample characteristics.

Characteristics		Total sample (N=17,676)	Western region sample (n=5884)	Central region sample (n=5150)	Eastern region sample (n=6642)	*P* value
Age, mean (range)		61.22 (45 to 108)	61.28 (45 to 97)	61.11 (45 to 108)	61.26 (45 to 95)	.48^a^
**Gender, n (%)**						.57^a^
	Male	8433 (47.71)	2785 (47.33)	2488 (48.31)	3160 (47.58)	
	Female	9243 (52.29)	3099 (52.67)	2662 (51.69)	3482 (52.42)	
**Education, n (%)**						<.001^a^
	Illiterate	4037 (22.84)	1516 (25.76)	1184 (22.99)	1337 (20.13)	
	≤Primary school	8150 (46.11)	2829 (48.08)	2246 (43.61)	3075 (46.30)	
	Middle school	3455 (19.55)	1001 (17.01)	1098 (21.32)	1356 (20.42)	
	≥High school	2034 (11.51)	538 (9.14)	622 (12.08)	874 (13.16)	
**Residency, n (%)**						<.001^a^
	Urban	7018 (39.70)	2200 (37.39)	1937 (37.61)	2881 (43.38)	
	Rural	10,658 (60.30)	3684 (62.61)	3213 (62.39)	3761 (56.62)	
**Marital status, n (%)**						<.001^a^
	Single	2437 (13.80)	1243 (21.13)	1074 (20.85)	1215 (18.29)	
	Partnered	15,237 (86.20)	4591 (78.03)	4076 (79.15)	5427 (81.71)	
Per capita income, median (range)		9427 (–175,000 to 3,028,000)	7690 (–175,000 to 1,889,710)	8596 (–74,600 to 1,880,000)	11,985 (–108,500 to 3,028,000)	<.001^a^
**Living near children, n (%)**						.14^a^
	No	8117 (45.92)	2677 (45.50)	2441 (47.40)	3075 (46.30)	
	Yes	9559 (54.08)	3207 (54.50)	2709 (52.60)	3567 (53.70)	
**Physical activity, n (%)**						<.001^a^
	No	1550 (8.77)	392 (6.66)	467 (9.07)	687 (10.34)	
	Yes	16,126 (91.23)	5492 (93.34)	4683 (90.93)	5955 (89.66)	
**Retirement, n (%)**						<.001^a^
	No	11,870 (67.15)	4066 (69.10)	3482 (67.61)	4287 (64.54)	
	Yes	5806 (32.85)	1818 (30.90)	1668 (32.39)	2355 (35.46)	
**Types of chronic diseases, n (%)**						<.001^a^
	None	3577 (20.24)	1023 (17.39)	980 (19.03)	1574 (23.70)	
	1 type	4201 (23.77)	1307 (22.21)	1275 (24.76)	1619 (24.38)	
	2 types	3600 (20.37)	1242 (21.11)	1053 (20.45)	1305 (19.65)	
	≥3 types	6298 (35.63)	2312 (39.29)	1842 (35.77)	2144 (32.28)	
**Drinking, n (%)**						<.001^a^
	No	11,622 (65.75)	3972 (67.51)	3376 (65.55)	4213 (63.43)	
	Yes	6054 (34.25)	1912 (32.49)	1774 (34.45)	2429 (36.57)	
**Smoking, n (%)**						.12^a^
	No	12,919 (73.09)	4295 (72.99)	3788 (73.55)	4775 (71.89)	
	Yes	4757 (26.91)	1589 (27.01)	1362 (26.45)	1867 (28.11)	
**Depressive symptoms, n (%)**						<.001^a^
	No	12,668 (71.67)	3843 (65.31)	3638 (70.64)	5187 (78.09)	
	Yes	5008 (28.33)	2041 (34.69)	1512 (29.36)	1455 (21.91)	

^a^Outcomes of the Kruskal-Wallis test. The regional division standard comes from the National Bureau of Statistics of the People’s Republic of China [68].

The proportion of respondents with a college education or above was higher in the eastern region (874/6642, 13.16%), while the highest proportion of illiterate respondents was observed in the western region (1516/5884, 25.76%). Conversely, the eastern region had a higher proportion of retired respondents (2355/6642, 35.46%), and the western region had the highest proportion of respondents with 3 or more chronic diseases (2312/5884, 39.29%). In terms of depressive symptoms, the western region exhibited a substantially higher proportion compared with both the central and eastern regions (western region: 2041/5884, 34.69% vs central region: 1512/5150, 29.36% vs eastern region: 1455/6642, 21.91%).

### Multidimensional Internet Use and Regional Differences Among Middle-Aged and Elderly People

Table 4 presents the details of internet use among respondents from different regions. Only a relatively small group (2395/17,676, 13.55%) reported using the internet. Among internet users, mobile phones held an overwhelmingly dominant position, surpassing other methods by a significant margin. The frequency of internet use varied, with respondents indicating either daily use (n=1991), infrequent use, or no use. Regarding the purposes of internet use, the majority of interviewees reported 3 main activities: chatting, watching news, and videos. A smaller number of interviewees played games or managed finances online. In the emerging internet landscape, mobile payment and mobile social networking played significant roles (*P*<.001 for both). Most participants used WeChat (n=2268), and a substantial portion of them also posted moments on WeChat (n=1701), indicating a desire for increased social interaction. Additionally, mobile payment tools (eg, WeChat Wallet or Alipay; n=1418) were frequently used to meet the convenience needs of the digital payment era.

**Table 4:** Multidimensional internet use and regional differences among middle-aged and elderly people.

Characteristics			Total sample (N=17,676)		Western region sample (n=5884)		Central region sample (n=5150)		Eastern region sample (n=6642)		*P* value
**Internet use, n (%)**											<.001^a^
	Yes	2395 (13.55)		622 (10.57)		740 (14.37)		1033 (15.55)			
	No	15,281 (86.45)		5262 (89.43)		4410 (85.63)		5609 (84.45)			
**Ways of internet use, n(%)**											<.001^a^
	Desktop computer	470 (2.66)		96 (1.63)		143 (2.78)		231 (3.48)			
	Laptop computer	134 (0.76)		31 (0.53)		47 (0.91)		56 (0.84)			
	Tablet	137 (0.78)		29 (0.49)		34 (0.66)		74 (1.11)			
	Mobile phone	2276 (12.88)		594 (10.10)		710 (13.79)		972 (14.63)			
**Frequency of internet use, n (%)**											<.001^a^
	Almost every day	1991 (11.26)		497 (8.45)		626 (12.16)		868 (13.07)			
	Almost every week	184 (1.04)		59 (1.00)		54 (1.05)		71 (1.07)			
	Not regularly	220 (1.24)		66 (1.12)		60 (1.17)		94 (1.42)			
	Never	15,281 (86.45)		5262 (89.43)		4410 (85.63)		5609 (84.45)			
**Purpose of internet use, n (%)**											<.001^a^
	Chat	1545 (8.74)		434 (7.38)		472 (9.17)		639 (9.62)			
	Watching news	1907 (10.79)		495 (8.41)		588 (11.42)		824 (12.41)			
	Watching videos	1535 (8.68)		416 (7.07)		489 (9.50)		630 (9.49)			
	Playing games	530 (3.00)		139 (2.36)		152 (2.95)		239 (3.60)			
	Financial management	152 (0.86)		33 (0.56)		41 (0.80)		78 (1.17)			
	Mobile pay	1418 (8.02)		344 (5.85)		468 (9.09)		606 (9.12)			
	WeChat use	2268 (12.83)		590 (10.03)		700 (13.59)		978 (14.72)			
	Post WeChat moments	1701 (9.62)		456 (7.75)		528 (10.25)		717 (10.79)			

^a^Outcomes of the Kruskal-Wallis test.

Compared with respondents living in the eastern and central areas of China, those in the western region not only had a lower proportion of internet use but also exhibited a more uniform pattern of usage, with fewer than 1% reporting the use of laptops (31/5884, 0.53%) and tablets (29/5884, 0.49%). However, a significant proportion (*P*<.001) of respondents in the western region engaged in various online activities, including using WeChat and posting moments, making mobile payments, chatting, watching videos, and reading news.

### Multidimensional Internet Use and Depression in Middle-Aged and Elderly People

Table 5 summarizes the relationship between internet use and depression from 3 dimensions: the devices, frequency, and purpose of internet use. The study also analyzed regional differences in internet use between the eastern, central, and western regions. For the overall sample, internet use was negatively associated with depression (odds ratio [OR] 0.613, 95% CI 0.542-0.692). No matter which terminal device was used to access the internet, depression was significantly reduced. Additionally, the higher the frequency of internet use, the lower the incidence of depression (infrequent: OR 0.631, 95% CI 0.482-0.817; frequent: OR 0.609, 95% CI 0.533-0.695). Regarding the purposes of internet use, except for financial management, all other purposes showed significance, reducing depression compared with the control group (chat: OR 0.667, 95% CI 0.578-0.767; watching news: OR 0.581, 95% CI 0.505-0.667; watching videos: OR 0.683, 95% CI 0.591-0.786; playing games: OR 0.593, 95% CI 0.458-0.757). In addition, mobile payment and mobile social networking significantly reduced the occurrence of depression (mobile payment: OR 0.583, 95% CI 0.495-0.683; WeChat: OR 0.616, 95% CI 0.543-0.697; OR 0.607, 95% CI 0.525-0.670).

**Table 5:** Internet use and depression in middle-aged and elderly people.

Characteristics			Total sample, odds ratio^a^ (95% CI)		Western region sample, odds ratio (95% CI)		Central region sample, odds ratio (95% CI)		Eastern region sample, odds ratio (95% CI)		
**Internet use (reference: no)**											
	Yes	0.613^b^ (0.542-0.692)		0.548^b^ (0.208-0.481)		0.608^b^ (0.488-0.753)		0.539^b^ (0.432-0.667)			
**Devices (reference: no)**											
	Desktop computer	0.536^b^ (0.392-0.719)		0.341^c^ (0.157-0.657)		0.543^d^ (0.314-0.886)		0.497^c^ (0.296-0.790)			
	Laptop computer	0.314^b^ (0.147-0.588)		0.153 (0.009-0.721)		0.251^d^ (0.060-0.706)		0.298^d^ (0.072-0.825)			
	Tablet	0.482^c^ (0.268-0.807)		0.689 (0.198-1.844)		0.318 (0.075-0.914)		0.310^d^ (0.107-0.711)			
	Phone	0.631^b^ (0.558-0.714)		0.574^b^ (0.457-0.715)		0.628^b^ (0.503-0.780)		0.549^b^ (0.438-0.682)			
**Frequency (reference: never)**											
	Regularly (almost every day/week)	0.609^b^ (0.533-0.695)		0.548^b^ (0.427-0.699)		0.598^b^ (0.472-0.752)		0.530^b^ (0.417-0.668)			
	Not regularly	0.631^b^ (0.482-0.817)		0.546^c^ (0.340-0.847)		0.662 (0.397-1.060)		0.626 (0.351-1.046)			
**Purpose (reference: no)**											
	Chat	0.667^b^ (0.578-0.767)		0.598^b^ (0.464-0.764)		0.667^c^ (0.515-0.856)		0.612^b^ (0.472-0.786)			
	Watching news	0.581^b^ (0.505-0.667)		0.567^b^ (0.441-0.724)		0.560^b^ (0.437-0.713)		0.481^b^ (0.358-0.637)			
	Watching videos	0.683^b^ (0.591-0.786)		0.628^b^ (0.484-0.808)		0.606^b^ (0.466-0.780)		0.677^c^ (0.523-0.866)			
	Playing games	0.593^b^ (0.458-0.757)		0.447^b^ (0.271-0.707)		0.583^d^ (0.361-0.904)		0.552^c^ (0.350-0.835)			
	Financial management	0.767 (0.466-1.202)		0.691 (0.230-1.688)		1.067 (0.507-2.082)		0.428 (0.128-1.066)			
	Mobile pay	0.583^b^ (0.495-0.683)		0.584^b^ (0.432-0.780)		0.494^b^ (0.370-0.652)		0.531^b^ (0.395-0.703)			
	WeChat use	0.616^b^ (0.543-0.697)		0.552^b^ (0.439-0.690)		0.584^b^ (0.465-0.729)		0.558^b^ (0.446-0.693)			
	Post moments	0.607^b^ (0.525-0.670)		0.497^b^ (0.381-0.643)		0.611^b^ (0.472-0.784)		0.560^b^ (0.431-0.718)			

^a^Odds ratio <1 indicates that internet use is negatively associated with depression, and the smaller the odds ratio, the stronger the protective effect.

^b^*P*<.001.

^c^*P*<.01.

^d^*P*<.05.

Comparing respondents in different regions, laptop use significantly reduced depression in eastern and central China (eastern: OR 0.298, 95% CI 0.072-0.825; central: OR 0.251, 95% CI 0.060-0.706). Tablet use was significantly associated with a reduction in depression among people in eastern China (OR 0.310, 95% CI 0.107-0.711). There was also a significant decrease in depression among daily and infrequent internet users in the western region (daily: OR 0.548, 95% CI 0.533-0.695; infrequent: OR 0.546, 95% CI 0.340-0.847).

### Analysis of Mediating Effect of Social Participation

#### Indirect Effects of Multigroup Pathway Analyses by Region

Table 6 shows the indirect effects of the multigroup pathway analyses by region, revealing that social participation had a significant (*P*<.001) partial mediating effect on the relationship between each dimension of internet use and depression.

**Table 6:** The indirect effects of multigroup pathway analyses by region.

Indirect effects of multigroup pathway analyses by region pathways		β Coefficient (bootstrapped 95% CI)	SE	*P* value
**Total sample**				
	Devices of internet use → Social participation → Depression	–0.170 (–0.209 to –0.127)	0.022	<.001
	Frequency of internet use → Social participation → Depression	–0.065 (–0.081 to –0.047)	0.009	<.001
	Purpose of internet use → Social participation → Depression	–0.043 (–0.053 to –0.031)	0.006	<.001
**Western sample**				
	Devices of internet use → Social participation → Depression	–0.192 (–0.269 to –0.122)	0.037	<.001
	Frequency of internet use → Social participation → Depression	–0.091 (–0.128 to –0.059)	0.018	<.001
	Purpose of internet use → Social participation → Depression	–0.056 (–0.079 to –0.036)	0.011	<.001
**Central sample**				
	Devices of internet use → Social participation → Depression	–0.151 (–0.217 to –0.088)	0.033	<.001
	Frequency of internet use → Social participation → Depression	–0.064 (–0.092 to –0.038)	0.011	<.001
	Purpose of internet use → Social participation → Depression	–0.043 (–0.062 to –0.025)	0.009	<.001
**Eastern sample**				
	Devices of internet use → Social participation → Depression	–0.112 (–0.165 to –0.059)	0.028	<.001
	Frequency of internet use → Social participation → Depression	–0.048 (–0.070 to –0.026)	0.011	<.001
	Purpose of internet use → Social participation → Depression	–0.032 (–0.047 to –0.017)	0.008	<.001

To be specific, the use of different devices (*P*<.001), the frequency of internet use (*P*<.001), and the purpose of internet use (*P*<.001) all had a significant effect in reducing the depression score among the middle-aged and elderly. For the total sample, the indirect effect (β) of social participation on devices of internet use and depression scores was –0.170, SE 0.022, *P*<.001, bootstrap 95% CI –0.209 to –0.127. The indirect effect (β) of social participation on the frequency of internet use and depression was –0.065, SE 0.009, *P*<.001, bootstrap 95% CI –0.081 to –0.047. Meanwhile, the indirect effect (β) of social participation on the purpose of internet use and depression was –0.043, SE 0.006, *P*<.001, bootstrap 95% CI –0.053 to –0.031.

#### Summary of Proportion of Mediating Effects

**Figure 2:**
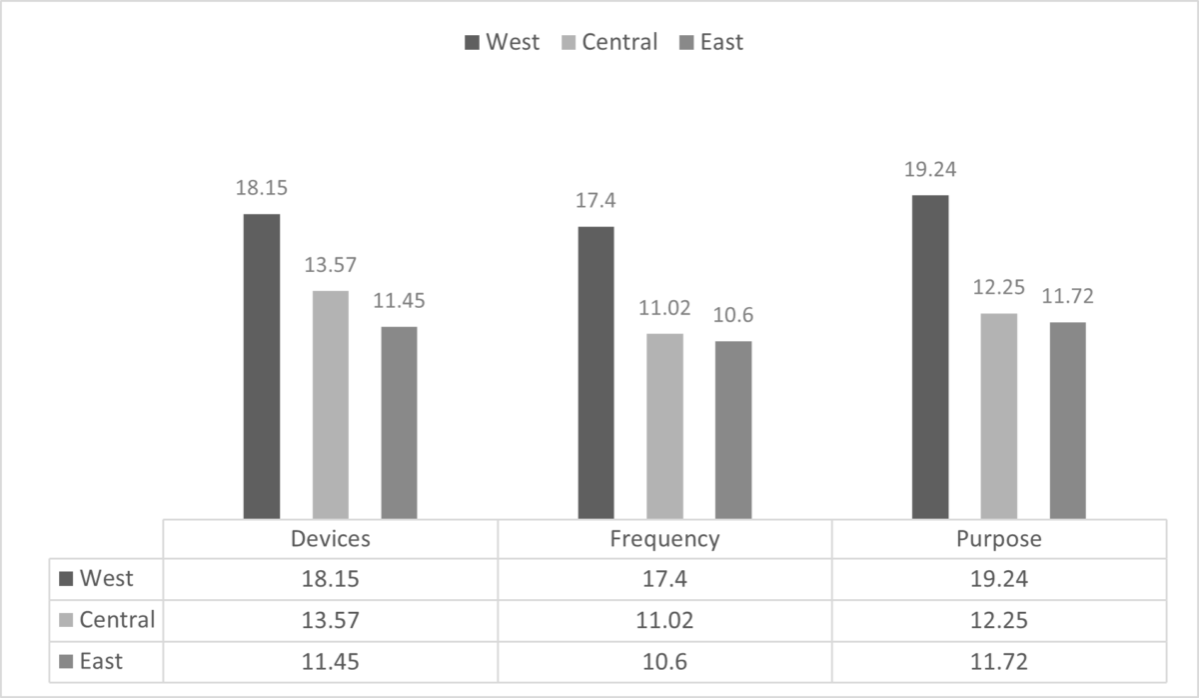
Comparison of the proportion of mediating effects among regional research objects.

In terms of devices, frequency, and purpose of internet use, the western region is higher than the central and eastern regions (Figure 2).

### Moderating Effect of the RIDL

Interaction test results are presented in Tables S1 and S2 in Multimedia Appendix 1. The moderating effects of the RIDL on internet use, social participation, and depressive symptom scores were tested using interactions between the RIDL and the dimensions of devices, frequency, and purpose of internet use. The results showed that in the relationship between internet use and social participation, the interaction between informatization development level and each dimension of internet use was statistically significant (internet use: *F*_74.12,9.82_=7.55, *P*<.001; devices: *F*_51.65/9.88_=5.23, *P*=.005; frequency: *F*_66.74/10.08_=6.62, *P*=.001; purpose: *F*_66.52/9.78_=6.80, *P*=.001). In the relationship between internet use and depression, the interaction between informatization development level and frequency of internet use was statistically significant (frequency of internet use: *F*_662.67/188.79_=3.51, *P*=.03).

Tables S3 and S4 in Multimedia Appendix 1 present the results of the simple slope tests for the different moderating paths. Based on the significant interaction, a simple slope test analysis was conducted to assess the influence of internet use on social participation under different echelons of informatization development. As a result, each echelon of the RIDL had a negative moderating effect on the relationship between various dimensions of internet use and social participation, as well as a negative moderating effect on the relationship between frequency of internet use and depressive symptom scores. Details are presented in Figures S1-S3 in Multimedia Appendix 1.

## Discussion

### Principal Findings

This study uses the latest data from the only publicly available database representative of middle-aged and elderly people in China, CHARLS, to explore the relationships among multidimensional internet use, social participation, and depression in this population. At the individual level, we first investigated the significant relationship among its 3 dimensions and depression. Then, we explored the partial mediating role of social participation between individual-level multidimensional internet use and depression. At the regional level, we confirmed the negative moderating effect of the RIDL on the relationship between individual-level internet use and both social participation and depression.

This study revealed a higher prevalence of depression (5008/17,676, 28.33%) among middle-aged and elderly Chinese individuals, which is consistent with some previous studies [56,66]. However, this prevalence is not only much higher than in some Asian countries [69] but also higher than in some Western countries [70]. This not only serves as a reminder of the dire situation China faces as the burden of depression increases but also highlights the urgent need for more widespread and feasible public interventions. Furthermore, the study observed significant differences in the prevalence of depression among residents in the eastern, central, and western regions, and the overall results showed a trend of “higher prevalence in the west compared with the east, with increasing prevalence from west to east,” which is consistent with previous research conclusions based on the Chinese background [71]. The reason may be attributed to China’s unique national conditions. Compared with the central and eastern regions, the western region is vast and sparsely populated, with a relatively low overall social development level, relatively backward infrastructure construction, a lower general social and economic status of the people, and deficiencies in social participation, among other factors, all of which may increase the risk of developing depression. These findings suggest that the mental health of middle-aged and older adults in the midwest region deserves special attention.

In the research sample, most middle-aged and elderly people did not use the internet (15,281/17,676, 86.45%). Although not using the internet remains the norm, middle-aged and elderly internet users are rapidly increasing due to the development of the internet and the impact of the COVID-19 pandemic. This trend aligns with data from the Ministry of Industry and Information Technology [72]. In terms of internet use patterns, there are significant differences between the eastern, central, and western regions.

Specifically, for the frequency and purpose of internet use, the majority of users access it every day. This could be attributed to the higher socioeconomic status and easier access to internet devices in the central and eastern regions, where digital life has become the norm. By contrast, for respondents in the western region only the use of mobile phones, as an internet device, was significantly associated with depression. This is likely due to the lower cost and higher value of mobile phones in the mobile internet era, making them more accessible. The study also found that a higher frequency of internet use was associated with a lower likelihood of developing depression. This is because daily internet use has become a normal part of life for middle-aged and elderly individuals. They use the internet for various purposes, such as seeking health information [73], chatting, watching the news, and making mobile payments [74]. All of these activities help them integrate into digital life without experiencing the negative effects of addiction. Among the purposes of internet use, only financial management did not show a significant association with depression. This could be because middle-aged and elderly people tend to be more conservative in financial management. However, other purposes of internet use significantly reduced depression symptoms, likely because the internet is not limited by time and space. The development of portable mobile online social networks has further increased opportunities for social contact, reducing the cost of social connection and enabling active participation in society, which enhances social integration [75]. Communication and exchanges with other older adults, friends, and especially family members help develop a sense of social connection and expand their social networks, ultimately reducing the likelihood of developing depression [76].

From the perspective of structural social capital, we have observed the following pathways of influence of internet use on depression in middle-aged and older adults. In general, social participation, as a major component of structural social capital, has a significant partial mediating effect on the relationship between multidimensional internet use and depression. It may be that multidimensional internet use enables cheap and easy communication between friends in distant communities with common interests [77], thus restoring a sense of community through both distant and close interpersonal relationships [46], increasing social connections, overcoming social and spatial barriers, and providing a convenient way to stay in touch with friends and the outside world, which, while increasing online social participation, also better enables offline social participation [78]. Social networks act as ties that provide the cohesion and security necessary to support mental health, and social engagement with a wider network improves mental health by conveying more diverse information [79]. Furthermore, the mediating effect generally shows a trend of “west > central > east.” This suggests that the internet can benefit those with fewer social resources, and engaging in a variety of internet activities is indeed an important complement to acquiring offline social capital gains [80]. The likely reason is that, in the context of the digitization of numerous resources, the internet, serving as a powerful empowerment tool [81], can not only alter the distribution and delivery of digital resources but also facilitate the connection between individuals and resources or others. For marginalized middle-aged and elderly people in the western region, the internet provides a valuable opportunity to utilize this empowerment tool to gain more resources and social connections [82]. In addition, internet use enables them to participate in more social activities; for example, the proliferation of internet use and social media platforms such as WeChat and Tik Tok can greatly enrich their lives, allowing them to benefit significantly from receiving external information, shopping, and entertainment [83], which, in turn, increases social connectivity and improves their well-being. Moreover, engaging in online social activities allows people not only to gather more information about offline social activities but also to seek and receive assistance from others, thereby enhancing self-efficacy, fostering a sense of self-control, and decreasing the likelihood of experiencing depressive states [84]. Therefore, increasing internet use among middle-aged and elderly individuals across different regions can serve as a way to compensate for the potential deficit in social capital.

Upon introducing the RIDL in an innovative manner, this study discovered a moderating effect on the relationship between individual-level internet usage and social participation. Surprisingly, the RIDL appears to weaken this relationship, which contradicts our initial hypothesis. This outcome may be attributed to the geographical location of the third echelon, which often comprises regions with lower levels of economic and overall development. Individuals within this echelon are more likely to face exclusion from internet use due to various social and economic factors, which in turn affects their level of social participation.

While sociodemographic conditions do play a role in internet use, factors such as availability, physical access, and sustained use contribute to the digital divide. This divide emphasizes disparities in user access to infrastructure, encompassing aspects of autonomy and continuous access [85]. For socioeconomically disadvantaged groups, acquaintances’ support and their expectations largely determine internet use, while self-efficacy is influenced by usage patterns and encouragement within social networks.

In this context, measures such as promoting basic digital infrastructure construction, advancing the informatization industry, and providing educational compensation, all of which are promoted by public organizations, can help facilitate the intelligent use of the internet among disadvantaged segments of society. This empowers them to explore resources for personal and recreational needs, expand ongoing learning opportunities [86], facilitate communication with family and friends [87], and improve access to social information, thereby increasing social participation.

In regions with relatively high levels of informatization development, residents exhibit higher levels of social participation and health information literacy. As a result, the marginal utility of further informatization development decreases, leading to a diminishing impact of individual internet use on social participation and the alleviation of depression.

### Study Contributions

This study makes several contributions to theory and practice. First, this study provides preliminary evidence in the context of China, where internet use among middle-aged and elderly people remains at a very early stage compared with those in developed countries. This study not only examines the direct relationship between multidimensional internet use and depression but also attempts to explore potentially important connections between these variables.

Second, this study is one of the pioneers in investigating how the RIDL moderates the relationships between individual internet use-social participation and internet use-depression. Previous research on internet use among middle-aged and elderly individuals often overlooked the influence of regional factors on the individual level. The heterogeneity of the benefits of internet use in different regions has been unclear until now. Our findings highlight that the internet tends to benefit disadvantaged groups more in regions with lower informatization development levels. These insights have meaningful implications, and we believe this area presents promising opportunities for future research to gain a deeper understanding of the interplay between technology-related divides at both the individual and regional levels.

Third, our research demonstrates that the internet holds promise as a potential auxiliary strategy in the digital age for middle-aged and elderly groups. This feasibility spans different resource settings and aims to reduce the exposure of vulnerable groups to the risk of adversity while enhancing protective factors to ensure that depression interventions are available to those who need them.

### Study Limitations

This study has several limitations. First, the use of the database is limited to the CHARLS survey. In addition, compared with other countries, the complexity and completeness of information on the internet in China are relatively simple; therefore, follow-up studies could consider additional databases, such as pooling databases from low- and middle-income countries, which might yield more representative research findings. Second, due to limited data availability, CHARLS only asked about the devices, frequency, and purpose of internet use, but it did not detail the specific time of use for each purpose. Therefore, our study could not discuss the existence of internet addiction. Third, questions related to internet use are self-reported by respondents, which may have problems with recall errors and the inability to accurately capture the frequency of internet use. Additionally, this study uses cross-sectional data, and the relationship between the 2 (ie, internet use and depression) is based on theoretical derivation, which cannot fully rule out possible endogeneity problems, such as reverse causality. The causal relationship between the 2 needs a more rigorous research design, such as the use of natural experiments, instrumental variables, or longitudinal data validation.

### Conclusions

This study offers preliminary evidence that different dimensions of internet use are associated with reduced depression, with social participation mediating the relationship between multidimensional internet use and depression among middle-aged and elderly Chinese within the context. Specifically, social participation partially mediates the association between multidimensional internet use and depression in the eastern, central, and western regions, respectively. In addition, the RIDL helps individuals engage more in internet use and social participation, thereby reducing the impact of depression. However, this effect weakens sequentially from the western region to the central region and then to the eastern region. This study suggests that efforts should be made to improve the availability of internet use and implement policies that support internet use by middle-aged and older people in different regions.

## Appendix

Multimedia Appendix 1 Interaction test results.

## Abbreviations

CES-D-10: 10-item Center for Epidemiologic Studies Depression Scale
CHARLS: China Health and Retirement Longitudinal Study
OR: odds ratio
RIDL: regional informatization development level

## Data Availability

The data that support the findings of this study are available from the China Health and Retirement Longitudinal Study (CHARLS) data repository [88].
