# Supplementary material for: Multidimensional Internet Use, Social Participation, and Depression Among Middle-Aged and Elderly Chinese Individuals: Nationwide Cross-Sectional Study
Source: J Med Internet Res. 2023 Aug 30;25:e44514. doi: 10.2196/44514 (PMC10500359; doi:10.2196/44514)
Supplement: Multimedia Appendix 1 [file jmir_v25i1e44514_app1.docx]

**Appendix Table 7.** Interaction of RIDL and Internet use on social participation

| Interaction | F | P-value |
| --- | --- | --- |
| Devices of Internet use ×RIDL | 5.23 | 0.005 |
| Frequency of Internet use × RIDL | 6.62 | 0.001 |
| Purpose of Internet use × RIDL | 6.80 | 0.001 |

**Appendix Table 8.** Interaction between RIDL and frequency of Internet use on depressive symptoms

| Interaction | F | P-value |
| --- | --- | --- |
| Frequency of Internet use ×RIDL | 3.51 | 0.03 |

**Appendix Table 9.** RIDL moderate Internet use-social participation simple slope test

|  | β | S.E. | t | P-value | [95% CI] |
| --- | --- | --- | --- | --- | --- |
| **Devices of Internet use** |  |  |  |  |  |
| First echelon | 0.337 | 0.025 | 15.322 | <0.001 | [0.329, 0.425] |
| Second echelon | 0.468 | 0.028 | 16.613 | <0.001 | [0.413, 0.524] |
| Third echelon | 0.489 | 0.031 | 15.765 | <0.001 | [0.428, 0.550] |
| **Frequency of Internet use** |  |  |  |  |  |
| First echelon | 0.162 | 0.012 | 13.117 | <0.001 | [0.138, 0.186] |
| Second echelon | 0.216 | 0.014 | 15.334 | <0.001 | [0.188, 0.243] |
| Third echelon | 0.222 | 0.015 | 14.626 | <0.001 | [0.192, 0.252] |
| **Purpose of Internet use** |  |  |  |  |  |
| First echelon | 0.109 | 0.007 | 15.166 | <0.001 | [0.095, 0.123] |
| Second echelon | 0.139 | 0.008 | 17.821 | <0.001 | [0.122, 0.156] |
| Third echelon | 0.144 | 0.009 | 17.821 | <0.001 | [0.129, 0.160] |

**Appendix Table 10.** RIDL moderate Internet use-depressive symptom simple slope test

|  | β | S.E. | t | P-value | [95% CI] |
| --- | --- | --- | --- | --- | --- |
| **Frequency of Internet use** |  |  |  |  |  |
| First echelon | -0.379 | 0.084 | -4.527 | <0.001 | [-0.543, -0.215] |
| Second echelon | -0.594 | 0.094 | -6.336 | <0.001 | [-0.778, -0.410] |
| Third echelon | -0.667 | 0.078 | -8.594 | <0.001 | [-0.820, -0.515] |


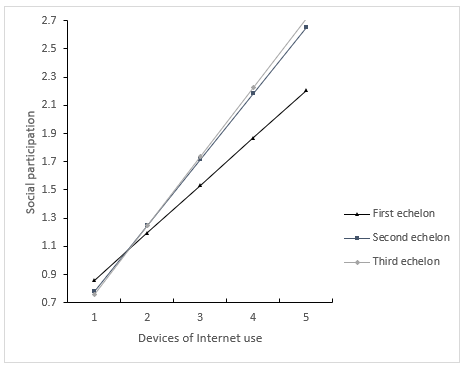


**Appendix Figure 3**. Interaction effects of RIDL in the relationship between Devices of Internet use and social participation


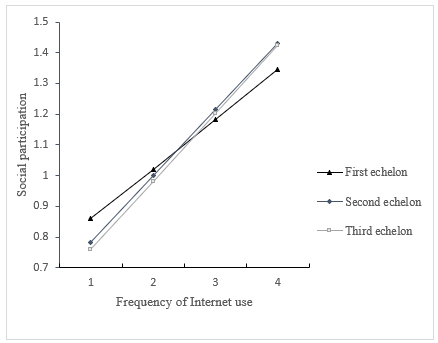


**Appendix Figure 4**. Interaction effects of RIDL in the relationship between Frequency of Internet use and Social participation


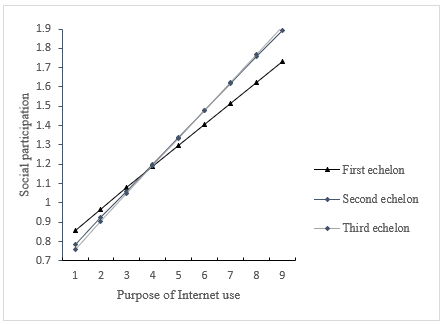


**Appendix Figure 5**. Interaction effects of RIDL in the relationship between Purpose of Internet use and Social participation

Table of China's East, Central and West regions

| Eastern region | Beijing、Tianjin、Hebei、Shanghai、Jiangsu、Zhejiang、Fujian、Shandong、Guangdong、Liaoning、Jinlin、Heilongjiang |
| --- | --- |
| Central region | Shanxi、Anhui、Jiangxi、Henan、Hubei、Hunan |
| Western region | Inner Mongolia、Guangxi、Chongqing、Sichuan、Guizhou、Yunan、Shaanxi、Gansu、Qinghai、Xinjiang |
